# Recontacting in clinical practice: the views and expectations of patients in the United Kingdom

**DOI:** 10.1038/ejhg.2017.122

**Published:** 2017-08-02

**Authors:** Daniele Carrieri, Sandi Dheensa, Shane Doheny, Angus J Clarke, Peter D Turnpenny, Anneke M Lucassen, Susan E Kelly

**Affiliations:** 1 Egenis, University of Exeter, Exeter, UK; 2 Faculty of Medicine, University of Southampton, Southampton, UK; 3 School of Medicine, Cardiff University, Cardiff, UK; 4 Royal, Devon and Exeter Hospital, Exeter, UK; 5 Wessex Clinical Genetics Service, University Hospitals Southampton NHS Foundation Trust, Southampton, UK

## Abstract

This paper explores the views and expectations of patients concerning recontacting in clinical practice. It is based on 41 semi-structured interviews conducted in the United Kingdom. The sample comprised patients or parents of patients: without a diagnosis; recently offered a test for a condition or carrier risk; with a rare condition; with a variant of unknown significance – some of whom had been recontacted. Participants were recruited both via the National Health Service (NHS) and through online, condition-specific support groups. Most respondents viewed recontacting as desirable, however there were different opinions and expectations about what type of new information should trigger recontacting. An awareness of the potential psychological impact of receiving new information led some to suggest that recontacting should be planned, and tailored to the nature of the new information and the specific situation of patients and families. The lack of clarity about lines of responsibility for recontacting and perceptions of resource constraints in the NHS tended to mitigate respondents’ favourable positions towards recontacting and their preferences. Some respondents argued that recontacting could have a preventative value and reduce the cost of healthcare. Others challenged the idea that resources should be used to implement formalised recontacting systems – via arguments that there are ‘more pressing’ public health priorities, and for the need for healthcare services to offer care to new patients.

## Introduction

The rapid accumulation of new genetic and genomic knowledge is resulting in better diagnosis and treatment of some health conditions. Patients seen by a genetics service may not have received a diagnosis in the past (due to insufficient knowledge of certain disorders) or may have had a diagnosis, but advances in medical science may bring new information as well as treatment or management options. This deluge of new clinical information is intensified by the increasing use of whole exome or genome approaches in healthcare, where variants of previously unknown significance (VUSs) may be reclassified as pathogenic or non-pathogenic.^1^ As more knowledge is accrued, the question of whether former patients should be recontacted, when updated information about their genetic variants is available, becomes more pressing.

There is a growing interest internationally in addressing the problem of recontacting – given the familial, predictive nature of genetic information and the fact that the significance of such information may change.^2, 3, 4, 5, 6^ However, as discussed elsewhere,^7, 8^ there is no consensus over whether and in what circumstances healthcare professionals (HCPs) should recontact patients, and there is also limited research on this topic.

Ethical arguments have been made in favor of recontacting, mostly on the ground that new genetic/genomic information can have significant implications for the health of patients and families, and their reproductive decisions, lifestyle choices, employment, and psychosocial well-being.^9, 10^ However, existing empirical evidence indicates that not all patients value recontacting and some would prefer not to be recontacted.^11, 12^ Recontacting may also affect patients negatively, causing anxiety and concerns over health and economic activity, and may be experienced as an intrusion into privacy and a violation of their interest^13^ or right not to know (RNTK).^14, 15^

A systematic review^11^ about recontacting points to a clash between the ethical desirability and the practical difficulty, due mostly to lack of resources, of recontacting. It also argues that, resources aside, there are many other aspects of recontacting that need investigation, for example, clarifying the situations in which recontacting is seen as a good standard of care, as well as role boundaries and responsibilities.

Some have argued that the task of recontacting should be performed by the medical specialist(s) who provides continuity of care to patients, which is often their general practitioner (GP).^10^ However, studies that explored patients’ and genetic HCPs’ views on recontacting found that both groups identified genetic HCPs as having responsibility to perform this task.^9, 16^ However, genetic HCPs tended to assign more responsibility to patients for maintaining contact with HCPs than patients.^9^ Some commentators have suggested that support groups – through regular newsletters and by posting news on their websites – may play a role in recontacting.^17, 18, 19, 20^ Our research has for the first time explored current practices and the views of HCPs and clinical scientists in the United Kingdom. We reported that recontacting was viewed as desirable under certain circumstances.^7, 8^ However, in line with the systematic review,^11^ there was a widespread concern about its feasibility due to insufficient resources and lack of clarity about role boundaries and responsibilities. Clarifying these issues was seen as a pre-requisite to the development of guidelines. The importance of bridging the ‘gap’ between the expectations of patients and those of HCPs was also highlighted.^7^ The current paper aims to address this gap by presenting the results from interviews with patients, which explored their views and expectations about recontacting.

## Methods

These interviews are part of a broad interdisciplinary study, which investigates clinical, ethical, legal and social issues related to recontacting in clinical genetics practice in the NHS in the United Kingdom (study website: http://ex.ac.uk.//mgc). The aim of these interviews was to investigate qualitatively patients’ expectations about how genomic information is managed and their expectations regarding responsibilities and mechanisms for recontacting. We were interested in the views of UK health service patients who had been, or potentially could be, recontacted. The study obtained approval from a NHS Research Ethics Committee; this allowed the recruitment of participants via regional genetics services, covering a combined population of around 8 million. Data collection took place between 2015 and 2016. Local collaborators at each site sent out study information and interested parties contacted the researchers directly. We did not ask them to record the number of patients they contacted, so we are unaware of our response rate. To broaden the sample, we recruited participants by posting information about our study on online condition-specific support groups. Participants were 41 patients or parents thereof. Conditions were self-reported: 18 had a condition that was rare (eg, myotubular myopathy) or undiagnosed; 11 had a suspected hereditary cancer or cardiac condition for which the genetic basis had not been found (ie, BRCA1/2-negative breast cancer, or a VUS); and 12 had a diagnosis that was clearer (eg, hereditary breast cancer or Fragile X). All were potentially ‘eligible’ for being recontacted - either for a test, a variant reclassification, or because a newly identified risk-reducing intervention was available. Four had been recontacted by the genetic service, who offered the patient a test where one was previously unavailable; four were recruited through support groups – none of these had been recontacted (for more details about the sample see Table 1). Interviews were semi-structured and face-to-face, except for two which were conducted by telephone. Questions were open-ended and investigated: experiences of genetic testing and/or having been recontacted by a HCP; potential expectations and preferences; and ideas about potential lines of responsibility. Interviews were recorded, transcribed verbatim, and subjected to thematic analysis.^21^ They were conducted and independently analysed by three members of the research team (co-authors 1–3). Key themes were identified and discussed in regular team meetings, and with co-authors. Some of the quotations used in this paper have been slightly edited for readability. Parents of patients were interviewed together and are ascribed a double hyphenated number, for example, P1-2. The interview guide is provided as Supplementary Information A.

## Results

### Pro-recontacting

The majority of respondents valued recontacting. New genetic/genomic information was generally linked to an improvement in the knowledge about a condition, or strategies of disease management or prevention – and was therefore seen to benefit both patients and family members.

*P2 Forewarned is forearmed isn’t it, because then, if there’s something preventative that you can do,* [it] *doesn’t rule you out from getting it but [...] your chances are better*

Some comments suggested an interpretation of recontacting as in line with a ‘long-sighted’ healthcare system – in which some resources are used to leverage medical innovation to prevent or reduce the severity of diseases (as opposed to a ‘fire-fighting’ approach in which resources are mainly used to treat overt manifestation or severe symptoms).

*P5 Healthcare is not just there at the point of crisis, that's the point of where you are in dire need and you need a lifesaving operation or it should look after you or it should be there, be available to you throughout being ill, preventing you being ill, recovering, and helping you live a healthy life beyond any illness*

It is important to highlight there were different degrees of endorsement of recontacting. Many respondents’ comments revealed not only a preference, but also an expectation to be recontacted in light of new information, and/or the suggestion of the concept of a professional responsibility to recontact patients.

*P40 If the information is there I think it should be given to somebody. I don’t think there’s any point in holding back*

*P7 I would definitely, a thousand percent want to know. […] I think you’ve got an obligation to pass this information on*

Some instead regarded recontacting as desirable, but not necessary; and others – like the respondent below – deployed a low degree of endorsement, appearing almost neutral about the utility of recontacting (see also ‘Resources’ section).

*P6 I wouldn’t object but I, I mean, I wouldn’t regard it* [recontacting] *as essential*

### Different thresholds of significance of new information

Respondents’ views about what kind of new information was significant, or would correspond to a professional responsibility to recontact varied. Most expressed the preference, or expectation, to be recontacted in relation to new information that is clinically significant/actionable and, crucially that is tailored specifically to them or their family. This example below from a respondent with cardiomyopathy is revealing:

*P23* [I would expect to be recontacted] *if there’s something significant that would be relevant to me and my particular condition. [...], but if it was just a case of, ‘Oh we’ve found a new gene that causes cardiomyopathy, we are just letting you know’, then no, I wouldn’t expect that*

However, some respondents – especially parents of children with genetic conditions – valued the possibility to receive any type of new information, irrespective of its clinical significance/actionability, or specificity. For this group, new medical information as such was associated with a greater chance of understanding and exerting control over one’s or a family member’s condition (eg, a more nuanced understanding of the causes, or getting closer to a diagnosis).

*P13-14 We’d rather know everything which would help the boys, we feel that the more you know about something… it’s better to prepare or understand, isn’t it?*

*P10-11 By greater understanding, it gives you a reason to know why it’s happening.… I’d rather that correct information comes to us as soon as they find it*

*P28 I think ignorance is not always bliss, is it? If something happened and we didn't know, we could have done something to prevent it…I think it's important to know if there's something that can be done to help, definitely*

Some respondents placed emphasis on having regular contact with the healthcare service, expressing eagerness to be notified also about the lack of new information. This preference – which stretches recontacting to a form of regular follow-up – gave voice to a perceived difficulty in managing the uncertainty (in terms of risk/diagnoses/prognoses) that genetic and genomic medicine can generate, and the related challenge of dealing with what was perceived as the slow pace of clinical practice and research.

*P12 We’ve learnt with genetics, ‘soon’ can mean anything between one and two years*

*P22 Even if it’s: ‘We haven’t found anything’ […] I would just like to know, because it’s just like a waiting, isn’t it? You just think ‘ooh, I wonder if they have found anything’ or ‘I wonder if anything has happened’ or ‘how’s it all going?’*

*P31 I’d like to be kept informed like annually maybe […] there is the element of support within information*

### The complexity of receiving new information

Respondents deployed diverse and nuanced accounts of the potential psychological effects of receiving new information. Some expressed their eagerness to receive updates even when new information is not linked to improvement of health outcomes.

*P34 I think knowledge is power. I would want to know. Even if it was really bad, I’d want to know. If they said ‘actually everyone who’s got this is going to be dead in ten years’, I’d want to know [...] and then I can live my life accordingly*

Others voiced concerns about the psychological impact that recontacting may have for both themselves, or other patients – in particular if the new information was perceived as not linked to an improvement of health outcomes.

*P5 I suppose being very blunt about it, if the information you are given leads to bad news, would you want to know that, do you know what I mean? I mean probably me, probably yes, but I could imagine a lot of people wouldn't want that*

Concrete examples of the complex psychological impact of receiving new information – irrespective of its perceived positive or negative value – were provided by the few respondents who had experienced being recontacted. The quotation below illustrates how a positive attitude towards being recontacted, and a feeling of gratitude, do not counter the potential psychological complexity of the experience.

*P20 It was only about three months ago. I wasn’t expecting a hospital letter. And it did throw me. […] I can’t explain why because to me it was brilliant, I was really glad that things had moved on, but it was still quite a shock that I thought, maybe I’d get an answer now [...] I had a long chat with* [consultant who recontacted respondent] *on the phone then before I went to see him. And I was honest with him, I said, ‘You’ve completely floored me’. It was just completely out of the blue, really, but in a good way*

Another important point made was that recontacting should be planned, according to the nature of the new information and the specific patient. This point concurs with the above-mentioned preference expressed by some respondents to receive bespoke information.

*P34 If it was a significant development, that was going to be very impacting or life-limiting, then I think that would need to be quite carefully managed in terms of how that information is given to people. It wouldn’t necessarily have to be a face-to-face appointment, [it] would just have to be quite carefully managed, which I have every faith that it would be*

An interesting corollary was the suggestion to ‘coordinate’ recontacting with timely access to the clinical service, to offer patients the opportunity to discuss the new information and its implications with HCPs. The quotation below vividly illustrates this point.

*P12 The worse you can have, is ‘Oh I’ve got information, but I can’t see you until two months’*

### Right not to know

The so called patient ‘right not to know’ (RNTK) was another contentious aspect related to the complexity of receiving new information. Some respondents argued that patients who have expressed a desire not to be recontacted should be recontacted anyway – especially if the new information is clinically relevant. This group placed more emphasis on HCPs’ duty of care and/or the utility of receiving new information, than on patients’ RNTK.

*P7 I think it’s a duty of care…whether they* [patients] *want to hear it or not sometimes you have to hear things you don’t want to know*

Others, while acknowledging the potential tension between HCPs’ duty of care and patients’ RNTK, emphasised the importance of respecting patients’ preferences. Like the respondent below, some added that this view presupposes that the preference is made in a condition of full capacity.

*P23 If the patient is mentally fit and has said they don’t want to hear anything further, then I think their wish should be respected because it’s up to them. Obviously, there will be exceptions to that in the case of people that aren’t capable of making decisions such as that, but if such as myself or my brother, if we said, ‘No, we don’t want to hear anything further’, then I would expect that to be respected.* […] *It’s a difficult one because obviously the responsibility is with the consultant, I suppose they would want to inform the patient, but if the patient has said they don’t want to know then they can’t force them to know*

Some expressed more nuanced compromises based on the suggestion that recontacting – and, in general, the HCP-patient relationship – should be seen as an open process in which both patients and HCPs have the possibility to get back in touch when appropriate. Again this suggestion stretches recontacting to a form of follow-up.

*P30 I think they should respect your decision, but maybe periodically ask if that is still the way you feel.*

*P37 I think they ought to leave the door open. They ought to be able to say to them, ‘Look I know you find this devastating today, but next week, when you’ve had time to think about it or calm down or talk to somebody else about it, you might want to come back to me’*

### Unclear responsibilities

Respondents found it challenging to link their recontacting preferences and expectations to ideas about roles and responsibilities. There was a general agreement that there should be no time limit for this responsibility. This reinforces the idea of the benefit of receiving updates presented above.

*P29 You can’t put a time limit on it, because we’re always going to be finding out something new about it, so no there shouldn’t, well why would there be a time limit, it seems a silly thing to put a stop on it. It’s like saying, oh, we’re going to stop the research now, it’s, what we know is all we want to know, we don’t want to know anymore*

There were however mixed and divergent opinions about who should be responsible for recontacting. A relatively common position was that genetic HCPs should be responsible, as they were identified as the specialty with the expertise to understand, and keep up to date with, developments in genomic medicine.

*P7 I think now genetics have stoked it all up, I think they have got a little bit of an obligation now to keep me in the loop*

Emphasising the logistical aspects of recontacting, some argued for the involvement of GPs, mostly on the grounds of being the speciality with the most up-to-date patient demographic and clinical information, and an efficient system that could be leveraged for recontacting purposes.

*P17 Perhaps by then people have changed the address or... and haven't contacted them* [genetic HCPs] *and let them know. I think if it's done through their GPs, their GP will always have an up to date address. If I let my GP know they'll always have my up to date address so then they'll always be able to get back to me on any new findings…because it could take years to find anything out*

Others placed emphasis on the HCP-patient relationship, arguing that ideally the HCPs who are involved in the management of the patient, or are more ‘familiar’ with the patient, should be those responsible for recontacting them. The quotation below illustrates this point and, drawing on the general decline of continuity of care within primary care in the NHS, it also challenges the idea that GPs should be involved in recontacting.

*P8 I don’t think it could be the GP. Twenty years ago when you had a family GP it would be different but we don't anymore. I see a different GP every time I go. I think it should come from not necessarily the person you saw because that might not be possible, but if possible the person that has been involved in your care. I think it should come from somebody that you are aware of already*

Along a similar line of argument, there were respondents who explicitly resisted the idea that one speciality should be responsible, claiming instead that this responsibility should be distributed across the HCPs and specialties involved in the management of the patient. Some extended this responsibility to patients – suggesting that patients could contact HCPs (genetic or other specialties involved) to ask for updates.

*P30 I think it’s everyone’s responsibility. But I think there needs to be some kind of mechanism there to bring everyone back together, you know periodically, to go over that.* [...] *The professionals have access to tools that we don’t, that give them information that we would never have access to, so that’s why it needs to be both*

As the quotation above shows, the argument of patients’ involvement was generally not framed as a complete transfer of responsibility for recontacting onto patients, but rather as a joint venture between patients and HCPs, enabled by some form of infrastructure or governance.^22^ This idea of joint venture was principally justified drawing on widespread discourses about each patient’s responsibility for managing their own health, and/or as being a sustainable way to enact recontacting within the resource-constrained NHS.^22^

There were contradictory views about a potential responsibility of support groups. Those in favour tended to use the following arguments: support groups may have more resources than the NHS to use for recontacting purposes, and they may be effective communicators of updates as they are more in tune with the psychosocial dimension of living with genetic conditions, as opposed to the biomedical approach of HCPs.^23^

*P13 I think they probably would be better at doing it because they also get funded and a lot of people raise money for them, so maybe you know they would be better to do it on their behalf*

*P38…the NHS is such a big organisation, it’s very clinical, where the patient support group is pertaining to what you’ve had or got or likely to get or it’s dealing with what you are sort of going through, so it’s a bit more personal. So yeah, I would think they have got a role*

The main arguments of those who were against were the opposite, that is, they revolved around the idea of the financial status and lack of resources of support groups, that medical updates should be communicated to patients by HCP, and that patients do not always want to join support groups.

*P2 I think there’s a place in society for them to support these people, but I think that information needs to come from somebody medically qualified …*

*P29 …They’re a charity at the end of the day and they could fold tomorrow. And it’s difficult for them, [...] and as the research gets bigger and bigger, they’re not going to be updating every newsletter, every month, every 6 months, they might only do one every 18 months or 2 years, which might be too late, as research moves on.*

Finally, it is important to point out that the general lack of clarity about recontacting responsibility appeared to be intensified by discourses of resource issues.

*P35 I know everybody’s busy in the NHS, but where does it stop? Do you start a new role?*

### Resources

An awareness of a lack of resources and cuts in the NHS challenged or weakened respondents’ recontacting preferences, expectations, and ideas about responsibilities. For example, many of those who favoured recontacting struggled to imagine its implementation, recognising that the sustainability of the NHS is already at stake with the current levels of services, staff, time, and other resources. Broad challenges were also recognised; the most common revolved around the idea that resources should be invested to tackle more urgent public health priorities (eg, diabetes, obesity). Another broad and related challenge mentioned was the priority to offer a service to new patients, before investing resources to recontact former patients:

*P1 It's just impossible isn't it, I don’t think they cope, I think they do a marvellous job but there's just not the resources there, are there, it's as much as they can do to cope with the new cases that keep coming along, never mind those that are in the past*

As the extract below illustrates, the awareness of resource constraints could influence the degree of endorsement of recontacting described in the section ‘Pro Recontacting’.

*P18 It* [recontacting] *would be helpful, yeah. If they've got the time and resources to do that then it doesn't matter*

Finally, there were respondents who attempted to find a compromise between their preferences and their perception of the current status of the NHS, suggesting models of recontact that would be more ‘basic’ and require fewer resources. For example, the passage below illustrates a proposal of a model of sustainable implementation in which the bespoke elements of recontacting are sacrificed.

*P5 I probably don't expect it because I think I've experienced how inundated healthcare professionals are in the day to day treatment of patients. [..] But I do think it would be helpful and beneficial if it could happen. But then it would probably have to happen on a general basis rather than on a very personal basis*

## Discussion

Overall, these findings indicate that patients tend to value the potential for recontact as an important means to bring the clinical and psychological benefits of biomedical research to families in a timely fashion. As in our study based on interviews with HCPs,^7^ there was a lack of clarity about lines of responsibility for recontacting and a tension between its desirability and its feasibility. The lack of clarity and the perception of resource constraints tended to weaken respondents’ favourable assessment of recontacting and to downgrade their expectations of it in practice. These assessments and expectations were also weakened in the light of ethical considerations relating to justice. Some respondents challenged the idea that resources should be used to implement recontacting, drawing on ethical arguments about the existence of more pressing public health priorities and the need for healthcare services to offer care to new patients. However, others claimed for recontacting a value in the prevention of disease or diminution of risk and, thereby, a potential reduction of healthcare costs. These individuals were therefore more in favour of its implementation in practice within the NHS. There were different views concerning the type of information patients would expect to receive in any recontact, in line with research on the personal utility of genetic information.^24, 25^ However, respondents’ accounts – for example, the ideas that HCPs should plan recontacting, and the suggestion that the HCPs ‘known’ to the patients and/or involved in the management of their condition should be involved – pointed to a recognition that in certain circumstances receiving new information may trigger complex psychological reactions, irrespective of how bespoke or actionable the new information might be. This recognition validates the suggestion that a discussion between HCPs and patients about recontacting in the context of consent for testing may be beneficial.^26^ Such discussion may help patients to reflect on the issue of the evolving nature of genomic information, adjust their expectations about the possibility of accessing new information (also in the light of what the healthcare service can offer) and express their preferences. It may also help the HCPs to gather some information that may enable them to offer a form of recontacting that would be in line with patients’ preferences, reducing the potentially negative psychological effects. Interestingly, the idea that GPs may be involved in recontacting was mostly justified on the ground of their patient records – rather than on the argument presented previously in the literature that they provide continuity of care.^10^ It appears unlikely that support groups could play a central role in updating patients about personally tailored genomic information, given their potentially precarious financial status, and that not all patients seen by HCPs may end up joining these groups.^27^ However, the question about whether they may play a role in relation to specific conditions remains open. Some respondents suggested that support groups may be in a suitable position to convey new information because more in tune with the psychosocial dimension of living with genetic conditions. This gives emphasis to the idea of the potential complex psychological impact of receiving new information.

There was an adequate understanding of what recontacting means, partly because the interviewers had the opportunity to explain and provide examples of recontacting scenarios during the interview. Nevertheless, despite the effort to frame the interviews as focusing on recontacting in clinical practice, some respondents tended to conflate the clinical and research dimensions. Moreover, some stated preferences (eg, to receive regular updates from HCPs) corresponded to a model of follow-up (ie, never being discharged from the service) rather than recontacting (ie, being contacted after being discharged). This raises the question of how recontacting should be defined. Otten *et al.*’s^11^ systematic review defines recontacting as the ethical and/or legal obligation to recontact former patients in light of new genetic findings. We follow this definition, although we have previously highlighted that there are important distinctions between ‘duty’ and ‘responsibility’.^7^ A related challenge is to clarify what ‘former patients’ means in practice. Recontacting suggests a new contact with a patient, previously discharged from care, due to the emergence of new information. This definition implies a difference between recontacting and follow-up. However, as this research shows, this difference may not be relevant to some patients and some HCPs who do not formally discharge their patients (eg, genetics/cardiology). The boundaries between recontacting and follow-up may also vary in other countries with different models or systems of healthcare. We suggest that it is important to agree an operational definition of recontacting that could be useful to HCPs, clinical scientists, and patients across different countries.

This study has some limitations. We did not include patients from all UK genetic services. Nonetheless, we do not claim our findings to be generalisable. Our findings are, however, ‘transferable’: we have made our research context explicit so that HCPs from any service, within the United Kingdom and elsewhere, can determine the extent to which the findings apply to them. A small proportion of respondents had been recontacted, and we think that it would be important to investigate potential differences between this group and those for whom recontact is a hypothetical issue. Interviews were undertaken in the United Kingdom, which has a National Health Service and a long-established clinical genetics service. However, rigorous investigations of views and expectations of patients from the United Kingdom could be effectively shared in the European context, at least with countries with clinical genetics services framed in a national health system.

## Figures and Tables

**Table 1:** Patient clinical profiles (* recruited through support groups)

*Identifier*	*Diagnosis*	*Recontacted in a clinical setting? (ie, contacted with new information or a new test after discharge)*
1	Hereditary breast cancer (*BRCA2*)	
2	Hereditary breast cancer (*BRCA2*)	
3	Possibly hereditary breast cancer (tested negative for *BRCA1* and *BRCA2)*	
4	Possibly hereditary bowel cancer (tested negative for Lynch syndrome associated genes)	
5	Possibly hereditary breast cancer (tested negative for *BRCA1* and *BRCA2*)	
6	Carrier of *BRCA* gene and parent of child with hereditary breast cancer	Yes – recontacted for genetic test when one was unavailable before
7	Possibly hereditary breast cancer (tested negative for *BRCA1* and *BRCA2)*	
8	X-linked myotubular myopathy	Yes - recontacted for genetic test when one was unavailable before
9	Parent of children with possible mosaic trisomy 16	
10-11	Parents of children with undiagnosed microdeletion syndrome	
12	Parent of children with undiagnosed microdeletion syndrome	
13-14	Parents of child with chromosome 17 duplication	
15	Fragile X	
16	Fragile X	
17	Parent of child with undiagnosed chromosome disorder	
18	Familial hypercholesterolemia	
19	Familial hypercholesterolemia	
20	X-linked myotubular myopathy	Yes - recontacted for genetic test when one was unavailable before
21	*Parent of deceased child with *Xq28* duplication	
22	Parent of child with brachytelephalangic chondrodysplasia punctata	
23	Possibly hereditary cardiomyopathy	
24-25	Parents of child with *PCDH19* epilepsy	
26-27	Parents of undiagnosed child	
28	Possibly hereditary hypertrophic cardiomyopathy (tested negative for main genes)	
29	*Parent of child with *1q21.1* microdeletion	
30	*Parent of undiagnosed child	
31	*Parent of a child with microdeletion as the probable cause of their neurodevelopmental problems	
32	Autosomal recessive Alport syndrome and parent of a child with Alport	Yes - recontacted for genetic test when one was unavailable before
33	Hereditary breast cancer (*BRCA1*)	
34	Possibly hereditary breast cancer (variant of unknown significance)	
35	Hereditary hypertrophic obstructive cardiomyopathy	
36	Possibly hereditary breast cancer (variant of unknown significance)	
37	Possibly hereditary ovarian cancer (variant of unknown significance)	
38	Possibly hereditary breast cancer (variant of unknown significance)	
39	Lynch syndrome	
40	Arrhythmogenic right ventricular cardiomyopathy (ARVC)	
41	Possibly hereditary breast cancer (tested negative for *BRCA1* and *BRCA2*)	
