# Supplementary material for: Recontacting in clinical practice: the views and expectations of patients in the United Kingdom
Source: Eur J Hum Genet. 2017 Aug 2;25(10):1106–12. doi: 10.1038/ejhg.2017.122 (PMC5602023; doi:10.1038/ejhg.2017.122)
Supplement: Supplementary Information [file ejhg2017122x1.docx]

# SUPPLEMENTARY INFORMATION

# recontact genomics, Interview with patients – draft V3

| **Themes** |
| --- |
| **Experiences** of genetic testing, having been re-contacted by HCP, got back in touch with HCP |
| **Expectations** about re-contacting: whether or not, who, when & **Preferences** about re-contacting methods |
| **Views** on **responsibilities** for re-contacting |

## Experiences

1. Could you tell me what is the health condition for which you or your family members are being seen?
   1. Can you tell me about your experience of your initial appointment in the clinical genetics department?
   2. Did you have a genetic test? Which healthcare professional ordered it?
   3. How – and by which healthcare professional – were the results communicated to you?
2. Do you normally look for information/updates relevant to your (or your family members’) health condition?
   1. If so, where do you get this information from?
   2. If you don’t, why is this case?

1. Has a healthcare professional ever suggested that you get back/keep in touch with them to check for updates about new genetic tests/information? Or to update them about any meaningful change in your life and/or the life of your family (e.g. a pregnancy)?
   1. If so, could you tell me what happened? E.g. was it through an informal communication or through a letter or other formal communication? How did you feel about being asked to do this?
   2. If not, why do you think this is the case?
2. Have you ever been asked by a healthcare professional if you would like to be re-contacted?
   1. To your knowledge, did they also take notes of your answer? How?
   2. If not, why do you think this is the case?
3. Have you ever been re-contacted by healthcare professionals in relation to new genetic information that was relevant to you or your family member(s)?
   1. If so, could you tell me what happened? Who contacted you? How did they contact you? What did they contact you about? How did you feel about being re-contacted?
4. Have you (or a family member) ever got back in touch with healthcare professionals to check for any new information relevant to your health?
5. If so, could you tell me what happened?
6. If not, why do you think this is the case?
7. Have you (or a family member) ever got back in touch with healthcare professionals to update them about any meaningful change in your life and/or the life of your family?
   1. If so, could you tell me what happened?
   2. If not, why do you think this is the case?

## Expectations and Preferences

1. Do you expect healthcare professionals to re-contact you if there are new developments (in relation to your genetic test results/genetic information) that could be relevant to your (or your family members’) condition?
   1. Do you think your family members would agree with you? Could you explain why?
   2. Do you think patients’ with your condition would agree with you? Could you explain why?
2. Do you have any personal preferences about how you would like to be re-contacted? (e.g., face to face meeting, personal letter, general letter, telephone call, email, Phone app, etc.) Why?
3. Are there any circumstances in which you would not like to be re-contacted? (e.g., you want to keep your health information private from family members, you prefer not to be reminded of your health condition in the future)
4. If so, could you give an example?
5. Do you think your family members would agree with you? Could you explain why?
6. Do you think patients’ with your condition would agree with you? Could you explain why?
7. If patients indicate they do not want to be re-contacted (or they do not want their family members to be re-contacted), do you think that there are any reasons a healthcare professional should re-contact them anyway? If so, could you give an example?

## Responsibilities

1. Do you think healthcare professionals should be responsible to update patients and their families about advances in genetics that may be relevant to their health?
2. If so, do you think there should be a time limit after which healthcare professionals are not responsible anymore?
3. In your view which healthcare professional should have this responsibility? Clinical genetics? Another healthcare professional you are seeing?
4. If not, why do you think this is the case?
5. Do you think that other groups, e.g., patient groups, should share this responsibility?
   1. If so, how? What role should they play?
   2. If not, why – in your opinion – should this not be the case?
6. Do you think that patients should also share this responsibility (for example by being responsible to check with the relevant HCP whether there are new updates on a regular basis)?
7. If so, how might this work?
8. If not, why – in your opinion –should this not be the case?
9. Do you think the NHS should offer a re-contacting service to patients?
   1. If yes, why? And how in your opinion would this work?
   2. If no, why not?
10. Is there anything you would like to add?

*Thank you for your time*
